# Recent Advances in (Bio)Chemical Sensors for Food Safety and Quality Based on Silver Nanomaterials

**DOI:** 10.17113/ftb.59.02.21.6912

**Published:** 2021-06

**Authors:** Irena Ivanišević, Stjepan Milardović, Petar Kassal

**Affiliations:** Faculty of Chemical Engineering and Technology, University of Zagreb, Marulićev trg 19, 10000 Zagreb, Croatia

**Keywords:** silver nanoparticles, nanosensors, chemical sensors, biosensors, food safety, food quality

## Abstract

There is a continuing need for tools and devices which can simplify, quicken and reduce the cost of analyses of food safety and quality. Chemical sensors and biosensors are increasingly being developed for this purpose, reaping from the opportunities provided by nanotechnology. Due to the distinct electrical and optical properties of silver nanoparticles (AgNPs), this material plays a vital role in (bio)sensor development. This review is an analysis of chemical sensors and biosensors based on silver nanoparticles with application in food and beverage matrices. It consists of academic research published from 2015 to 2020. The paper is structured to separately explore the designs of two major (bio)sensor classes: electrochemical (including voltammetric and impedimetric sensors) and optical sensors (including colourimetric and luminescent), with special focus on the type of silver nanomaterial and its role in the sensor system. The review indicates that diverse nanosensors have been developed, capable of detecting analytes such as pesticides, mycotoxins, fertilisers, microorganisms, heavy metals, and various additives with exceptional analytical performance. Current trends in the design of such sensors are highlighted and challenges which need to be overcome in the future are discussed.

## INTRODUCTION

Today, the food industry is the largest manufacturing sector and, at the same time, causes major global concern (*1*). Adulteration of products with low quality ingredients, or spoilage during transport and storage periods can easily affect the condition and quality of food. Improper use of pesticides and fertilisers can lead to contamination and, along with natural toxins, drugs, foodborne pathogens and heavy metals, can have a direct negative impact on human health. Besides possible harmful pollutants in food products, there are numerous compounds whose concentrations are of interest for nutritional food quality and, hence, need to be analysed. Thus, ensuring food safety and quality is of global importance (*2*).

The high complexity of food matrices sets a large challenge before scientists to develop reliable analyses of chemical and/or biological substances in trace quantities, thereby commonly requiring a variety of analytical methods to be employed. Conventional methods, such as distinctive chromatographic techniques, often coupled with mass spectrometry or enzyme-linked immunosorbent assay (ELISA) technique, possess the merits of sensitivity and accuracy. However, they often require complex sample pretreatment, sophisticated equipment and long analysis time, making them impractical for in-the-field food-specific applications. Chemical sensors and biosensors, low-cost devices that allow facile and quick determination of target analytes, are therefore being increasingly leveraged in the food sector (*3*).

Nanotechnology has brought a revolution in modern science, opening new possibilities for much needed improved food sensor technologies (*4*, *5*). Conjunction of nanoparticles with electrochemical or optical transducers has created new opportunities in small molecule detection, enabling sensor miniaturisation and fabrication on an industrial scale. Thus, nanotechnology offers cheaper, more reliable, quicker and highly sensitive nanocomposite architectures for application in the food industry. The immense leap in the application of nanotechnology is mainly attributable to advances in nanoparticle synthesis techniques during the last two decades, especially promoting silver nanomaterials (*6*).

Silver nanoparticles (AgNPs) are on the forefront of nanosensors, due to their distinctive localised surface plasmon resonance (LSPR) effect, combined with unique thermal and electrical properties. The colour change between dispersed and aggregated nanosilver grains can be associated with the concentration change of a target molecule in a suspension. This is a key feature in the fabrication of optical sensors (*7*). Furthermore, silver nanoparticles have been successfully paired with other nanomaterials in order to obtain novel hybrid architectures with enhanced electrochemical sensing performances. Nanosilver-based optical and electrochemical sensing arrays have found versatile application in the analysis of food and beverage matrices.

**Fig. 1:**
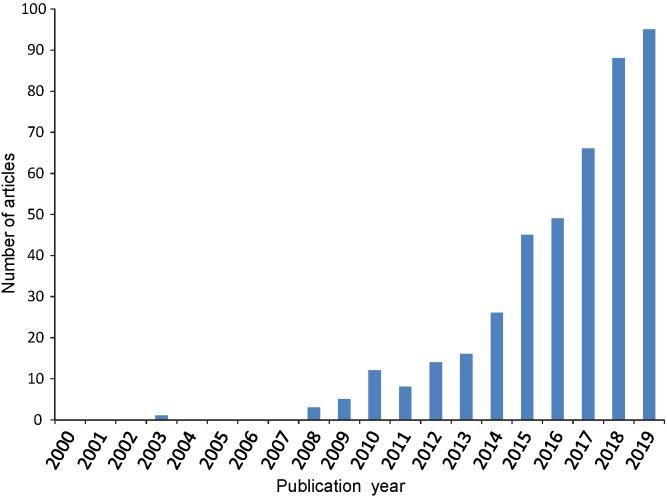
Number of publications per calendar year related to electrochemical and optical nanosensors in food, as determined by search in WoS platform (key words nano* AND *silver OR Ag AND *food AND *sensor)

A large number of original scientific papers (*8*-*13*), as well as review articles (*14*-*19*) regarding food quality and/or food safety topics have been reported recently, providing a broad survey trough the field of nanomaterial-based sensing platforms. In most of the publications, nanogold stands out above other metallic nanoparticles (*20*, *21*) and, although nanosilver-based devices are very abundant in the literature, a systematic review of food sensors based on silver nanoparticles has not been conducted (*22*-*24*). In the light of this, the authors considered the need to summarise selected examples of exclusively nanosilver-based (bio)chemical sensors for the detection of compounds concerning food safety and food quality. A literature survey using the Web of Science database revealed a large number of articles focused on these silver nanoparticle-based sensors for food matrices in the period of 2015–2020. The increasing number of publications in the selected period (Fig. 1) reinforces the importance of silver nanomaterial for food sensing purposes. The aim of this paper is to present the current state of knowledge and to give a critical review of the selected publications. This article is divided by transduction mechanism into two sections with the most frequent nanosilver-containing sensor categories – electrochemical and optical, preceded by a brief revision of nanosilver preparation approaches. In addition to electrochemical and optical sensors, our literature search has returned numerous examples of sensors based on surface-enhanced Raman scattering (SERS), exhibiting remarkable analytical performance (*25*-*31*). However, SERS sensors require highly sophisticated dedicated instruments and we have therefore omitted them from this review, focusing instead on sensors more appropriate for simple, rapid and on-site food analyses.

## SYNTHESIS OF SILVER NANOMATERIALS

**Fig. 2:**
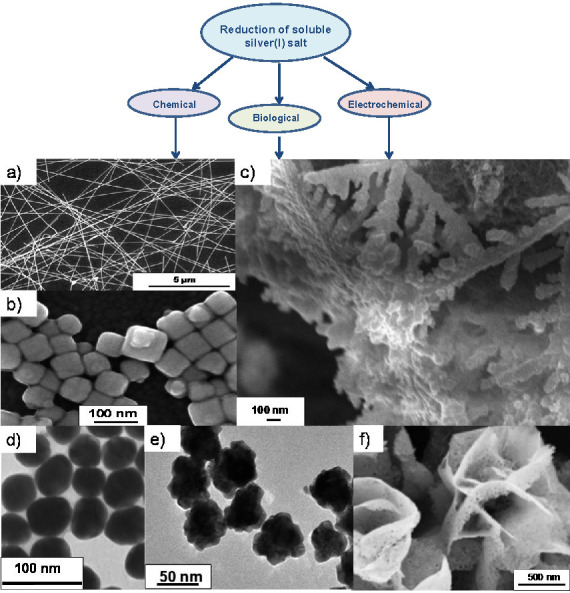
Schematic presentation of bottom-up synthetic routes from selected publications, combined with various nanosilver structures: a) nanowires (adapted from Min *et al.* (*32*) under CC BY 4.0 license), b) nanocubes (adapted from Hasna *et al.* (*33*), c) nanodendrites (adapted from Zhang *et al.* (*34*) under CC BY 4.0 license, d) nanospheres (adapted from Wang *et al.* (*30*) under CC BY-NC 3.0 license), e) roughly shaped particles (adapted from Fu *et al.* (*31*) under CC BY 4.0 license and f) nanoflowers (adapted from Huang *et al.* (*35*) under CC BY 4.0 license)

In general, synthetic routes of silver nanoparticles follow either bottom-up or top-down approaches. Top-down techniques are based on breaking down the bulk material by applying various physical forces; thereby allowing fabrication of small particles with a narrow size distribution without the involvement of hazardous chemicals. However, imperfections in the obtained surface structures and high energy consumption during the synthesis limit their broad application. The bottom-up approach uses silver atoms as elementary building blocks for controlled building-up assembly into a nanostructured material. In terms of particle synthesis for practical sensing application, applied bottom-up methods have the same denominator – reduction of a soluble silver(I) salt. The corresponding process can be conducted using appropriate chemical or biological reducing agent or aided by some external source such as electrical current (Fig. 2) (*30*-*35*). This is the basic principle of chemical, biological and electrochemical preparation methods (*36*). In the papers analysed in this review, synthesis of AgNPs was conducted exclusively through these bottom-up approaches. While the chemical reduction step was previously commonly performed using potentially hazardous agents (*e.g*. hydrazine (*37*) or hydroxylamine (*38*)), we have observed in this review a trend promoting green synthesis. The reduced silver nanomaterials are stabilised using a compulsory stabiliser molecule or capping agent; this is a key element that controls the particle size and shape. By modifying the reducing and capping agent and optimising the synthetic route, versatile silver nanostructures have been obtained and used in sensors (Fig. 2). As shown in this review, the type of silver nanostructure often has a large effect on the performance of the (bio)sensor in which it is employed. For the functional design of both electrochemical and optical sensors presented in this review, a powdered nanosilver product is often used. In these cases, the dried particles were either purchased (*39*, *40*), or isolated from the reaction suspension applying centrifugation (*41*, *42*), magnet separation (*43*), electrodeposition (*34*, *44*) or solution drying techniques (*45*, *46*). Furthermore, crystalline nanosilver product can also be obtained by implementing precipitation agents (*35*, *47*). Recently, we have shown that weak organic acids (*48*), as well as strong mineral acids (*49*), can be successfully applied to precipitate polyacid-stabilised nanosilver grains as the functional component of printable conductive inks.

## ELECTROCHEMICAL SENSORS

An electrochemical sensor is a compact analytical device in which an electrode is used as the transducer element (*50*, *51*). Regarding the way in which the transduction process occurs, electrochemical sensors can be categorised as potentiometric, voltammetric or amperometric and impedimetric. Unlike optical sensors, they can be easily used in turbid specimens (*52*).

The performance of all electrochemical sensors is strongly influenced by the working electrodes. The desired redox reaction of the analyte at the bare electrode often involves slow electron transfer kinetics and, therefore, occurs at potentials substantially higher than its thermodynamic redox potential. To overpower this obstacle, a redox mediator is often added into the measurement scheme. The mediator acts as a signal transducer between the analyte and the electrode. Nano-engineered silver material has a great potential as a modifier due to excellent electrical conductivity and high catalytic activity. Furthermore, composites made of pure silver nanomaterial embedded into polymer matrices or core-shell bimetallic structures render a combination of useful properties, offering remarkable prospects in the construction of modified working electrodes. For this purpose, nanostructured silver is a material of great importance for the development of electrochemical sensors (*53*).

**Fig. 3:**
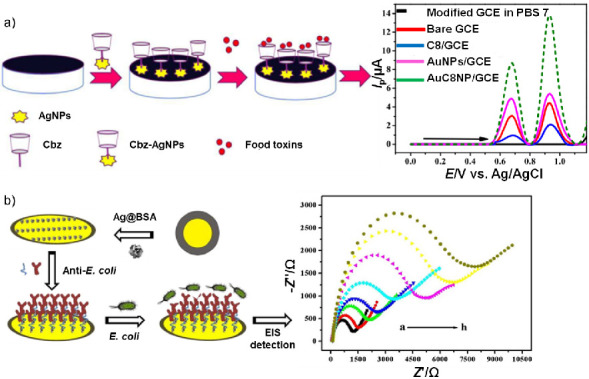
Selected examples of nanosilver-based (AgNPs) electrochemical sensors for food safety assurance: a) voltammetric sensor: carbamazepine (Cbz) coated silver nanoparticles glassy carbon electrode (GCE) based sensor for detection of Orange II (OR II) and Rhodamine B (RB). Binding of toxic food colourants results in two separate oxidation peaks at 0.67 V (OR II) and 0.93 V (RB) when measured by square wave voltammetry (schematic courtesy of A. Shah), and b) impedimetric immunosensor based on gold electrode coated with bovine serum albumin-conjugated silver nanoflowers (Ag@BSA) for selective detection of *Escherichia coli* O157:H7. Bacteria captured *via* antibody cause increment of impedance amplitude. Figure adapted from Huang *et al.* (*35*) under CC BY 4.0 license. PBS=phosphate-buffered saline, EIS=electrochemical impedance spectroscopy

A current trend in the sensor field is directed towards solving analytical problems with the development of cost-effective, miniaturised and portable devices that could be operated in the field (*54*). A survey of the silver nanomaterial-based electrochemical sensors for food applications published in the last five years is listed in Table 1 (*32**,**34**,**35**,**39**,**40**,**44**,**45**,**47**,**55**-**85*). Selected examples of electrochemical sensors, both voltammetric and impedimetric, are shown in Fig. 3 (*35*).

**Table 1:** Electrochemical sensors based on silver nanomaterials

Analyte	Sample	Analytical method	Recognition element	Nanomaterial	AgNP synthesis	LOD	Ref.
Nitrite	Water	Amp	Direct sensing	AgNP/MWCNTs	Electrodeposition	0.095 µM	(*55*)
Nitrite	Tap water	Amp	Direct sensing	AgNS	Green synthesis	0.031 µM	(*45*)
Nitrite	Milk, salami, mineral water	Amp	Direct sensing	rGO/AgNPs	Borohydride reduction	0.012 µM	(*56*)
Phosphate	Water	CV	Direct sensing	AMT/AgNW	Aldehyde reduction	3 µM	(*57*)
Monocrotophos, chlorpyrifos	Fruit samples	DPV	AChE enzyme	AgNP-N-F-MoS_2_ nanosheets	*In situ* reduction	0.2 pM (M) 3.0 pM (C)	(*58*)
Paraoxon	Garlic, cabbage	Amp	AChE enzyme	AgNP/AChE	–	4·10^–9^ ppb	(*39*)
PCB28	Tap water, guava juice	SWV	Anti-PCB	AgNP/GA/Ab	Citrate reduction	0.063 ng/mL	(*59*)
Pendimethalin, Ethyl parathion	Mineral and tap water, lettuce, honey	SWV	Direct sensing	AgNP	Borohydride reduction	36 nmol/L (PDM) 40 nmol/L (EPT)	(*60*)
Paraoxon	Onion	DPV	Direct sensing	AgNP	Electrodeposition	0.1 nM	(*61*)
Methyl parathion	Cabbage, green beans, strawberry, nectarine	Amp	Direct sensing	Ag@GNR	*In situ* reduction	0.5 nM	(*62*)
Thiodicarb	Soya milk	SWAdSV	Direct sensing	Ag nanopowder	Purchased	7.2·10^–9^ M	(*40*)
Vanillin	Cookie, pastry, jelly, chocolate	Amp	Direct sensing	Ag-Pd/GO	*In situ* reduction	5 nM	(*63*)
Sudan IV	Buffer	CV	Direct sensing	Au-Ag nanocomposite	Hydrothermal method	4 µM	(*64*)
Sudan I	Ketchup, chilli powder	Amp	Direct sensing	Ag-CuNP/rGO nanocomposite	Borohydride reduction	0.4 nM	(*65*)
Orange II, Rhodamine B	Water, fruit juice	DPV	Direct sensing	Cbz-AgNP	Borohydride reduction	1.2 nM (OR II) 1.0 nM (RB)	(*66*)
Amaranth	Buffer	LSV	Direct sensing	graphene/TiO_2_-AgNP	Ascorbic acid reduction	10^–7^ M	(*67*)
Sunset Yellow	Soft drinks	LSV	MIPs	GO/AgNP	Citrate reduction	0.02 µM	(*68*)
Chloramphenicol	Honey Milk powder	LSSV	Direct sensing	Ag nanodendrites/Short-MWCNTs-COOH	Electrodeposition	0.049 µM	(*34*)
*S. aureus*	Water	DPV	Aptamer	Apt/*S. aureus*/apt-AgNP complex	Borohydride reduction	1.0 CFU/mL	(*69*)
*L. monocytogenes*	Milk	Amp	Anti-*Lm*	Ag@[Ru(bpy)_3_]^2+^/chitosan	Borohydride reduction	2 cell/mL	(*70*)
Ascorbic acid	Orange, kiwi and apple juice	SWV	Direct sensing	AgNP	Green synthesis	0.1 µM	(*71*)
Ascorbic acid	Pimento juice, orange juice	SWV	Direct sensing	AgNP	Green synthesis	0.02 µM	(*72*)
Vitamin C	Fruit juice	CV	Direct sensing	AgNP	Citrate reduction	0.2 mM	(*73*)
Ascorbic acid	Vitamin C tablet	DPV	Direct sensing	Q-AgNPs-GNs	Electrodeposition	0.39 mg/mL	(*74*)
Hydrogen peroxide	Apple juice	Amp	Direct sensing	rGO-Nf@Ag	Hydrothermal method	5.35·10^–7^ M	(*75*)
Hydrogen peroxide	Milk	Amp	Direct sensing	Pd@Ag/rGO-NH_2_	Borohydride reduction	0.7 µA	(*76*)
Tyramine	Banana	Amp	Direct sensing	TiO_2_-Ag/Ppy	–	2·10^–8^ M	(*77*)
Urea	Milk, tap water	CV	Direct sensing	Ag-N-SWCNTs	Thermal synthesis	4.7 nM	(*78*)
Histamine	Fish sauce	DPV	Direct sensing	Ag-Ag_2_O/MWCNTs	Electrodeposition	0.18 µM	(*79*)
Ochratoxin A	Grape juice, wine	DPV	MIPs	AgNP/POM/rGO	*In situ* reduction	1.6·10^–11^ M	(*80*)
Aflatoxin M1	Milk	LSV	Direct sensing	GQD-*α*-CD-AgNP	Electrodeposition	2 µM	(*44*)
Microcystin-LR	Water	Amp	Anti-MC-LR	Ag@MSN	*In situ* reduction	0.2 ng/mL	(*81*)
Acetamiprid	Cucumber, tomato, wastewater	Impedance	Aptamer	Ag/NG	One-step thermal treatment	3.3·10^–14^ M	(*82*)
Bleomycin	Milk	Impedance	Aptamer	AgNCs/Apt@ CuFe@FeFe	*In situ via* aptamer template	0.0082 fg/mL	(*47*)
Ampicilin	Milk	Impedance	Aptamer	AgNP	Commercial ink	10 µg/mL	(*83*)
*E. coli*	Buffer	Impedance	Anti-*E.coli*	Ag@BSA nanoflowers	Ascorbic acid reduction	100 CFU/mL	(*35*)
*E. coli*	Eggshell, tap water	Impedance	Direct sensing	E.coli/PDDA/AuNP@Ag	*In situ* reduction	500 CFU/mL	(*84*)
*S. aureus*	Milk	Electrical conductivity	Direct sensing	HCR templated AgNWs	*In situ* reduction	50 CFU/mL	(*85*)
Hydrogen sulfide	Chicken	Electrical conductivity	Direct sensing	AgNW	Commercial AgNW solution	–	(*32*)

AgNP=silver nanoparticles, LOD=limit of detection, Amp=amperometry, MWCNT=multi-walled carbon nanotube, AgNS=silver nanospheres, rGO=reduced graphene oxide, CV=cyclic voltammetry, AMT=ammonium molybdate tetrahydrate, AgNW=silver nanowire, DPV=differential pulse voltammetry, AChE= acetylcholinesterase, PCB=polychlorinated biphenyls, GA=glutaraldehyde, Ab=antibody, SWV=square wave voltammetry, GNR=graphene nanoribbon, SWAdSV=square wave adsorptive stripping voltammetry, CuNP=copper nanoparticles, GO=graphene oxide, Cbz=carbamazepine, LSV=linear sweep voltammetry, MIP=molecularly imprinted polymers, LSSV=linear sweep stripping voltammetry, Apt=aptamer, Anti-*Lm=Listeria monocytogenes* antibody, Bpy=bipyridine, Q=quercetin, Nf=nafion, Ppy=polypirrole, SWCTN=single-walled carbon nanotube, POM=polyoxometalate, GQD-*α*-CD=graphene quantum dots-*α*‐cyclodextrin, MC-LR=microcystin-LR, MSN=mesoporous silica nanoparticles, NG=nitrogen doped graphene, BSA=bovine serum albumin, PDDA=poly(dimethyldiallylammonium chloride), AuNP=gold nanoparticles, HCR=hybridisation chain reaction

### Voltammetric sensors

Selective redox behaviour of an analyte of interest on the working electrode surface generates the current output, which is the basic principle of voltammetric sensor operation. During the measurement, the voltage of the working electrode can be changed linearly, in pulses or in cycles within a short time period, and the current produced by the system is measured. Detection established on the evaluation of current change at constant potential is also possible, which represents the basis of amperometric sensing. Operating in this mode, detected currents can be averaged over longer time periods, allowing more precise quantitative evaluation (*86*). The reviewed voltammetric sensors for detection of small molecules and chemical pollutants in food samples rely on the nanosilver-enhanced electron transfer processes of the modified working electrodes.

Fertilisers, including nitrite and phosphate anions, are common inorganic pollutants found in drinking water, soil and food. Controlling nitrite concentration is of significant importance, because its presence in the human body can cause conversion of haemoglobin into its non-oxygen carrier form, methaemoglobin (*41*). Usage of amperometry as a transduction pathway is the common denominator in nitrite-sensing devices, where selective redox behaviour is achieved at a single operating potential. Synergistic effect of AgNPs and multi-walled carbon nanotubes (MWCNTs) magnifies a glassy carbon electrode (GCE) working area, rendering favourable analytical performance towards nitrite oxidation (*55*). AgNPs/MWCNTs/GCE successfully produced a rapid signal output with continuous nitrite additions, which makes the proposed method suitable for nitrite determination in tap water. Another sensor including glassy carbon as an electrode material was proposed by Shivakumar *et al*. (*45*). An eco-friendly synthetic approach, using paper industry waste material, was developed to fabricate particles with average crystallite size of 30 nm. Amperometric experiments, conducted in 0.1 M phosphate buffer solution at a constant potential of +0.86 V, confirmed remarkable electrocatalytic properties of silver nanospheres towards nitrite oxidation. Simplicity in device fabrication, along with the green synthetic approach and high analyte selectivity, highlights this sensor for practical in-field application in water samples. Successive application of vacuum filtration and electropolymerisation was carried out to prepare a graphene-based/silver nanoparticle/poly(pyronin Y) hybrid paper electrode (*56*). High absorption coefficient of poly(pyronin Y) (poly(PyY)) decreases the electrooxidation potential of nitrite and expands the active electrode surface area, while silver nanoparticles enhance low electrical conductivity of the reduced graphene oxide (rGO). Flexible and free-standing rGO/AgNPs/poly(PyY) paper is the first reported graphene paper substrate for nitrite detection presented in the literature. Ammonium molybdate tetrahydrate (AMT)/silver nanowires (AgNWs) modified screen printed carbon electrode (SPCE) was employed in phosphate detection (*57*). The presence of one-dimensional nanowires causes faster electron transfer between the AMT and the SPCE, resulting in a significant increase in current response (fivefold) compared to the AMT/SPCE electrode.

Pesticides, by definition substances used to control pests, are xenobiotic compounds of utmost importance in the food safety sector (*87*). They can be classified by target organism (herbicides, insecticides, fungicides) or broadly by chemical structure (organic, inorganic, synthetic and biological), but mostly are grouped into organochlorines, organophosphates and carbamate families. Due to their large-scale use in agriculture, pesticides are the most abundant environmental pollutants. The presence of pesticide residues can seriously threaten human health and environmental safety, so continuous control of even low concentrations of pesticides in food-specific applications is mandatory (*88*).

First stage monitoring of pesticides in food and beverage matrices employed the usage of biosensors based on enzymes or antibodies as a recognition element. Acetylcholinesterase (AChE) is the most common enzyme used in electrochemical detection of organophosphorus and carbamate pesticides (*89*). These contaminants can cause irreversible esterase inhibition in the human central nervous system, leading to health issues. For selective electrochemical detection of monocrotophos and chlorpyrifos insecticides, a GCE modified with nitrogen-fluorine co-doped MoS_2_ monolayer decorated with AgNPs was proposed (*58*). To construct an effective biosensor, amino-functionalised carbon nanotubes (CNTs-NH_2_) were chosen for enzyme immobilisation onto the sensing platform, ensuring a high enzyme-to-substrate affinity (low Michaelis-Menten constant of 42 µM). Both cyclic voltammetry (CV) and electrochemical impedance spectroscopy (EIS) evaluation confirmed that the introduction of AgNPs has improved electron transfer kinetics and expanded the active surface area tenfold compared to bare GCE. Differential pulse voltammetry (DPV) responses of the AChE/CNTs-NH_2_/AgNPs-N-F-MoS_2_/GCE biosensor displayed linear decrement of the oxidation current with the increase of pesticide concentration, in accordance with the enzyme inhibition mechanism. The viability of this method was proven by selective pesticide determination in fruit samples. Zheng *et al*. (*39*) designed another AChE enzymatic sensor through chitosan layer-coated flexible nanosilver electrodes. The current of thiocholine (TCh) oxidation, as a product of the enzymatic reaction for indirect paraoxon detection, exhibited twofold better sensitivity when using an electrode made of nano- than microscaled silver powder. Feasibility of the proposed method in the analyses of vegetables, room temperature operation, as well as simplicity of fabrication through an eco-friendly approach, assert performance of this biosensor for practical purposes. Similar to pesticides, polychlorinated biphenyls (PCBs) are persistent organic pollutants known by their toxic health effects, once widespread for industrial purposes (*90*). Antibodies as a recognition element were successfully immobilised on the AgNPs/PANI/GCE *via* glutaraldehyde linker in a immunosensor designed for PCB 28 detection (*59*). Square-wave voltammetry (SWV) generated a linear electrochemical response to PCB 28, but also provided a signal for benzyl chloride and PCB 180 interfering agents. Another slight immunosensor disadvantage is the antibody immobilisation period (30 min) and PCB 28 incubation period (2 h), which can be further reduced by the usage of modified printed electrodes.

In response to enzyme instability and denaturation during storage, the second stage of pesticide monitoring resulted in the development of non-enzymatic sensors based on various silver-nanomaterial modifiers. Using chitosan-stabilised AgNP-modified GCE, the sensitivity of CV and adsorptive SWV methods in sensing the ethyl parathion (EPT) and pendimethalin (PDM) was compared by de Lima *et al*. (*60*). The aforementioned sensor was successfully applied for the detection of these two distinctive contaminants in diverse matrices – nitroaromatic herbicide (PDM) in mineral water, and typical organophosphate pesticide (EPT) in honey and lettuce samples. The accumulation step in voltammetric detection surpassed the problem of steep cathodic current decrement observed after successive CV cycles, thus lowering the detection limit and broadening the linear range. Among electrochemical detection methods, this modified electrode was the only one able to determine both pesticides. Moreover, the sensor has practical applications in various food matrices without requirement for the sample pretreatment step. In another study of paraoxon pesticide determination in onion samples, a stearic acid/nanosilver composite decorated GCE was used (*61*). Unlike the aforementioned biosensor-based device (*39*), the combination of biocompatible stearic acid and the electrocatalytic behaviour of AgNPs have shown a synergistic effect, thus enabling the development of an enzyme-free sensor. Differential pulse voltammetry was proven a satisfactory measurement technique, achieving the lowest paraoxon LOD value (0.1 nM) presented in the literature. Combining the AgNP electrocatalytic property with the oxygen-rich edge chemistry of a graphene nanoribbon platform, Ag@GNRs modified screen printed carbon electrode (SPCE) sensor was designed for selective methyl parathion (MP) pesticide determination (*62*). The lowest value of charge transfer resistance (71.4 Ω), obtained at the interface of Ag@GNRs/SPCE and electrolyte, compared with unmodified SPCE (460.1 Ω), GNR/SPCE (223.6 Ω) and Ag/SPCE (189.0 Ω), indicates synergistic interaction between the highly conjugated graphene-like material and AgNPs, which greatly reduces the overpotential and enhances the sensitivity towards MP. Well-defined amperometric responses for MP determination in cabbage, green beans, strawberry and nectarine were observed, with working range covering nano- and micromolar regions for each real sample. A novel voltammetric sensor, AgNP-supported solid amalgam electrode (SAE) for thiodicarb insecticide detection, was presented for the first time by Lucca *et al.* (*40*). Despite the inclusion of mercury, due to miniscule amounts involved, amalgam electrodes (*91*) adhere to the green chemistry approach. A CV study, recorded with AgNPs–SAE in Britton-Robinson buffer solution (pH=6.0), along with SWAdS voltammograms, revealed irreversible thiodicarb behaviour with a pronounced cathodic current peak at a potential of –0.64 V (*40*). Furthermore, linear dependence on the thiodicarb concentration from 1.05·10^–7^ to 1.52·10^–6^ mol/L, with 7.2·10^–9^ limit of detection, surpassed the lowest achieved values among all reported methods. Taking into account that this is only the fourth scientific publication regarding electrochemical methods for the voltammetric determination of thiodicarb, it is inevitable to emphasise this work as an esteemed scientific contribution.

Additive is a term which refers to chemicals used as adulterants for enhancement of the visual appeal or taste of some food products. Due to the toxic impact of some additives on human health, sustainable methods for efficient and rapid detection of these contaminants are necessary (*92*). Vanillin is an aromatic compound extensively used as a flavouring and fragrance enhancer, but high exposure to vanillin can cause liver and kidney damage (*93*). Li *et al*. (*63*) synthesised bimetallic Ag-Pd nanoparticles utilising green and *in situ* chemical reduction strategy, and demonstrated that the electrochemical response to vanillin at the Ag-Pd/GO/GCE sensor takes place at a lower potential than GCE modified with monometallic counterparts (Ag or Pd). DPV revealed the outstanding catalytic ability of the 3D nanohybrid material, enabling quantitative detection of vanillin at a concentration range of 0.02 to 45 µM. Azo-dyes (*e.g*. Sudan class of molecules, Orange II, Sunset Yellow) account for 60-70% of all synthesised colourants in the food industry. These compounds, characterised with at least one –N=N– functional group, are classified as carcinogens (*94*). GCE is the most important working electrode for selective determination of these food adulterants, due to its efficiency, accuracy and ability to enlarge the active surface area with various modifiers. Pani *et al*. (*64*) compared the sensitivity of AuNP, AgNP and Au-Ag core-shell composite material-decorated GCE, as a rapid voltammetric sensor for Sudan IV dye. Joining a green synthetic approach with the autoclave technology, obtained nanostructures were of the same composition, structure and properties in all batches of production. Hence, all three electrodes were found to be applicable in Sudan IV sensing, with current peaks proportional to dye concentration in practically the same linear range. GCE, modified with Ag-Cu nanoparticles anchored on reduced graphene oxide platform, was applied for determination of Sudan I in ketchup and chili powder (*65*). Amperometric current-time curves recorded in phosphate buffer solution (pH=6.5) at a constant potential of –0.112 V demonstrated the effective catalytic property of Ag-Cu/rGO/GCE reaching the lowest LOD value reported. Another food dye sensor was fabricated by drop-casting carbamazepine-functionalised silver nanoparticles (Cbz-AgNPs) onto the GCE (*66*). The bridging role of AgNPs leads to faster electron transfer between the donor (dye molecule) and acceptor (GCE), which is evident in the ability of the nanocomposite to boost the oxidation signals of Orange II and Rhodamine B dyes exceptionally, as compared to individual carbamazepine (Cbz)- or AgNPs-coated GCE (Fig. 3a). Achieved LOD values in the nanomolar range displayed high efficiency of this voltammetric sensor for the simultaneous detection of food dyes. Amaranth dye was chosen as an azo-dye model to study the electrochemical behaviour and degradation process utilising three graphene/TiO_2_–Ag nanocomposite-coated gold electrodes (denoted Au/GTA-5/10/15, regarding mass fraction in % of TiO_2_–AgNPs) (*67*). Linear sweep voltammetry (LSV) displayed the same obtained LOD value for all three electrodes (10^–7^ M), pointing out Au/GTA-10 in terms of considerably higher sensitivity towards the analyte. The same electrode exhibited first-order kinetics of amaranth degradation (2·10^–5^ M in 0.2 M KCl solution), tested by electrochemical polarization at +1.4 V *vs* Ag/AgCl. Molecularly imprinted polymers (MIPs) have been reported as excellent recognition elements for electrochemical sensors. An innovative sensor based on graphene oxide- and AgNPs-enhanced GCE was prepared using imprinting technology, with Sunset Yellow as the template molecule (*68*). Evaluated in the presence of Tartrazine, Amaranth, Brilliant Blue G and ascorbic acid as interfering agents, this sensor exhibited great selectivity towards the azo-dye, and was successfully applied for detection in soft drinks. GCE was also the working electrode of choice in the only example of chloramphenicol (CAP) detection in honey and milk powder samples (*34*). Decorated with Ag nanodendrites (immense specific surface area) anchored on carboxylic short-chain MWCNTs (fast electron transfer), the obtained sensor enabled ultrasensitive detection of CAP by linear sweep stripping voltammetry (LSSV) and CV techniques.

In the food safety sector, presence of pathogen bacteria needs to be strictly monitored. In order to detect *S. aureus* in water samples, bioassay system has been developed (*69*). Authors used two specific anti-*S. aureus* aptamer sequences. Primarily one, immobilised on streptavidin-coated magnetic beads, served as a capture probe, and the second one, conjugated to AgNPs, was the signalling probe. In the presence of target bacterium, a sandwich complex is formed which, after dissolution in 0.1 M HNO_3_, during anodic stripping DPV measurement produces distinctive AgNPs signal, sufficient to detect only one colony forming unit (CFU) in mL sample. *L. monocytogenes*, Gram-positive rod-shaped foodborne bacteria, have been successfully captured *via L. monocytogenes* antibodies attached to silver-ruthenium bipyridine complex core–chitosan shell hybrid nanoparticles (HNPs), chemically deposited onto GO nanosheets (*70*). At the applied potential of +0.55 V oxidation of bimetallic complex occurs, with measured change in amperometric response being directly proportional to the bacterial concentration.

Ascorbic acid (AA) is one of a few permitted substances in food and beverages (*95*). Due to its instability in acidic media, and upon oxygen/light exposure, AA degrades, which distorts food quality control *via* a colour change (*96*). Voltammetry is an increasingly popular method carried out in the analysis of AA in food samples, due to its simplicity alongside little or no sample preparation requirement. Diverse modifications of carbon paste electrodes have shown to stand out among other presented sensing tools in electrochemical AA detection (*97*). Applying a green synthetic approach through onion (*71*) and fig (*72*) extracts, Khalilzadeh’s group prepared AgNPs modifiers for fabrication of silver-carbon paste working electrodes (AgNPs/CPE). A simple sensor design, combined with SWV detection, revealed good selectivity for AA analysis in fruit juices, covering micromolar concentration ranges. Another simple sensor preparation for quick in-field vitamin C quantification in commercial fruit juices was described by Jadav *et al*. (*73*). Alternately adding carbon and silver conductive layers, authors fabricated the AA sensing area of silver/carbon SPE. In another study, coupling the electrodeposition of AgNPs (8 cycles in continuous cycling intervals from –0.7 to 1.9 V in 1.0 mM AgNO_3_ nitric acid solution), and quercetin from 0.5 mM solution in 0.1 M phosphate buffer (12 cycles of 0–40 mV potential scans), onto the graphene nanosheet-coated GCE, a new AA sensor was developed (*74*). DPV method provided three distinguished anodic peaks at the potentials of 10, 240 and 344 mV, corresponding to the simultaneous electrochemical oxidation responses to AA, uric acid and l-cysteine, respectively. Hydrogen peroxide is one of the most commonly used oxidising agents for the prevention of grocery spoilage. Thus, design of novel sensors for peroxide trace analysis is indispensable in the food quality sector. The rGO/AgNPs nanoarchitecture, coated onto the GCE *via* a Nafion layer, provided for the first enzymeless electrochemical selective detection of H_2_O_2_ (*75*). Using amperometry, the symbiotic effect of individual ternary hybrid nanostructure components significantly reinforced the sensor performance, enabling quantification of H_2_O_2_ in apple juice. A similar sensor, leaning on amperometry, GCE coated with a Nafion layer and reduced graphene oxide, this time modified with bimetallic Pd and Ag nanoparticles, was fabricated by Guler *et al.* (*76*). Due to the high loading and uniform dispersity of the prepared nanomaterial, the novel Nf/Pd@Ag/rGO-NH_2_ architecture showed noticeably improved catalytic properties towards H_2_O_2_. Unlike the previous reported sensor (LDR 1–10 μM), this sensor covers noticeably broader concentration ranges (2 to 19 500 μM).

In food quality analysis, freshness tests are based on detection of biogenic amines. In order to produce enzymatic or non-enzymatic amine sensors, adjustment of the GCE sensing surface with diverse nanostructured silver materials increases its practical analytical performance. Pioneering work in non-enzymatic sensing based on the TiO_2_-Ag/PPy nanocomposite material for amperometric tyramine (TA) detection in banana samples was done by Erdogan *et al.* (*77*). Under the optimum conditions (0.1 M phosphate buffer solution and the potential of +0.6 V), linearity over the 10^–8^–10^–6^ M concentration range, and LOD value lower than other TA detecting devices (2·10^–8^ M) revealed this nanocomposite-gelatin-coated GCE as an exemplary amine sensor. In a report by Kumar and Sundramoorthy (*78*), GCE coated with AgNPs-decorated nitrogen-doped SWCNT embedded in a Nafion layer (NF/Ag-N-SWCNT/GCE) served as a non-enzymatic sensor applicable for voltammetric urea detection in milk and water matrices. Butwong *et al*. (*79*) applied Ag-Ag_2_O-decorated MWCNT-modified GCE for the detection of histamine (HIS) in fish sauce. Based on the oxidation of the –NH_2_ group to the corresponding nitro compound during DPV measurements, the performance of the sensor was more stable and sensitive towards HIS (higher peak current shifted to a lower potential) than the CV data. As a result, determination of HIS, as a food spoilage indicator, at low concentration of 2 μg/L was achieved.

Mycotoxins are common food pollutants produced by organisms from the Fungi kingdom (*98*). Selective determination of mycotoxin in grape juice and wine samples was carried out applying MIPs (*80*). In this voltammetric sensor design, the GCE surface was coated with AgNPs, polyoxometalate (POM) and reduced graphene oxide layer, after which CV was used to imprint ochratoxin A (OCH). Synergistic effect between the AgNPs and POMs increased the rate of electrochemical reaction. Steep DPV troughs manifested ultra-high sensitivity of the MIP sensor towards OCH, with a detection limit of 1.6·10^–11^ M. For detection of aflatoxin M1 (AFM1) in milk samples, a modified GCE with long-term stability has been reported (*44*). The multilayered modifier film combines the advantages of α‐cyclodextrin (excellent electrical conductivity), graphene quantum dots (as mediator), and AgNPs as electrocatalytical agent for selective LSV recognition of AFM1. This sensor provided linearity from 0.015 to 25 mM concentration range. To develop a precise nonenzymatic immunosensor for electrochemical biosensing of cyanotoxin microcystin-LR (MC-LR), Zhao *et al*. (*81*) introduced silver@mesoporous silica (Ag@MSN) nanoparticles as a horseradish peroxidase-mimicking enzyme. Such engineered nanomaterial catalyses the reduction of hydrogen peroxide to produce a current signal inversely proportional to the MC-LR concentration. The biosensor performance manifests in a three orders of magnitude linear range, with a remarkable LOD value obtained in water samples (0.2 ng/mL), which is much lower than the concentration of 1 μg/L in drinking water, submitted by the World Health Organization (*99*).

### Impedimetric sensors

The application of electrochemical sensors based on impedance analysis has grown during the past decade owing to rapid response and the high sensitivity of such devices (down to picomolar range). The role of AgNPs is signal enhancement, which makes this technique extremely useful to detect the analyte of interest during interactions with (bio)sensing platforms. Despite this fact, extensive sensor development has been limited mostly by the complexity of impedance analysis. Hence, in this article only a few impedimetric sensors have been reviewed regarding both quality and safety sectors.

As artificially synthesised oligonucleotide or peptide molecules, aptamers found their versatile purpose as highly selective recognition elements for target molecules in impedimetric sensors. An AgNP (large surface area)–nitrogen-doped graphene (excellent electrical properties)–aptamer system has shown to be an effective biosensing platform for impedimetric acetamiprid detection (*82*). Obtained impedimetric data displayed a linear picomolar concentration range, resulting in the lowest LOD value for pesticide sensing (3.3·10^–14^ M) among all presented electrochemical sensors.

Antibiotics are a broad family of chemically synthesised compounds used as a cure for bacterial infections. However, their intense usage in medicine, but also as feed additive, has led to negative effects on animal and human health. The omnipresence of antibiotics in meat and dairy products is a hot topic attracting the public attention nowadays. Therefore, the trace antibiotic levels in food products need to be strictly regulated (*100*). Combining Prussian blue (FeFe-PB) core with bimetallic CuFe shell, and coupling with silver nanoclusters *via* aptamer linkage in a one-step bio-inspired synthesis, an impedimetric aptasensor for bleomycin (BLM) antibiotic was developed (*47*). Owing to the formation of Fe(II)·BLM complex, the AgNCs/Apt@CuFe@FeFe aptasensor outperformed the CuFe@FeFe-based one, giving an extremely low detection limit of 0.0082 fg/mL towards BLM. Rosati *et al.* (*83*) inkjet-printed an aptamer-functionalised sensor for ampicilin detection in milk samples. Although impedance data display a linear range over more than 2 orders of magnitude, the LOD value is not sufficient for prescribed EU standards. Nonetheless, this preliminary work presents a simple and cheap microelectrode fabrication method which can be easily subjected to further sensor improvement.

The combination of two distinctive silver nanomaterials with electrochemical impedance spectroscopy (EIS) detection technique have been shown as a rapid and efficient way for detection of *E. coli* in water and eggshell matrices. In the first report, bovine serum albumin templated 3D Ag nanoflower impedance immunosensor was fabricated (*35*). Pathogen capturing has been conducted *via* antibody binding and the charge transfer resistance (*R*ct) value increased proportionally to the logarithm of 3.0·10^2^–3.0·10^8^ CFU/mL concentration range (Fig. 3b). High specificity of the modified Au electrode was proven towards *C. sakazaki*, MRSA, *S. albus*, *L. easei* and *S. flexneri*. In the second report, encapsulation of negatively charged *E. coli* surface with positively charged poly(dimethyldiallylammonium chloride) (PDDA) enabled the connection between bacteria and nanogold particles (*84*). Silver enhancement reaction resulted in the formation of *E. coli*/PDDA/AuNP@Ag complex, which improved the performance of a novel microfluidic chip for the impedimetric detection of bacteria. The prepared complex increased the solution conductivity and the double layer capacitance around the microelectrodes, showing practical application in eggshell solution and tap water specimens.

A novel biosensor based on hybridisation chain reaction (HCR) as an enzymeless strategy for rapid bacteria detection in milk samples was proposed (*85*). In the presence of the biomarker (highly specific fragment of *S. aureus* 16S rRNA), and with the aid of hairpin-decorated gold nanoparticles, silver nanowire formation between the adjacent interdigitalised electrodes occurs. The switch from isolated AuNP nucleation sites to the conductive silver feature leads to the electrochemical signal transduction pathway. Despite the higher achieved LOD value (50 CFU/mL) than the previously presented *S. aureus* immunosensor analogue (only 1.0 CFU per mL sample) (*69*), the authors would like to highlight the benefit of the HCR method as a polymerase-free detection strategy, as well as the merits of high sensitivity and fast response (analysis accomplished in less than 100 min), which can be useful for point-of-care applications. Combining silk fibroin with silver nanowires, interesting biocompatible, wearable and optically transparent flexible bioelectronics were prepared (*32*). A single-use food sensor can be easily embedded into the vinyl or plastic food packaging. H_2_S produced during chicken spoilage induces Ag surface corrosion, decreasing its electrical conductivity through the formation of sulfide film. This strategy can be also applied in colourimetric sensor design (see following chapter).

## OPTICAL SENSORS

**Fig. 4:**
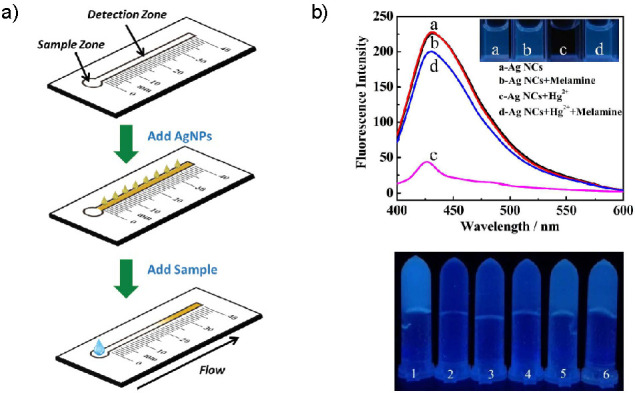
Selected examples of optical chemical sensors for food safety: a) schematic design of the colourimetric distance-based paper sensor for chloride ions. Reproduced from Phoonsawat *et al.* (*113*) with permission from The Royal Society of Chemistry, b) fluorescent sensor for melamine. On the top are fluorescence spectra and corresponding photographs under UV light demonstrating solution-based sensing; on the bottom are AgNC-doped agarose hydrogels under UV light (1 Ag NCs, 2 Ag NCs-Hg^2+^ system, and 3-6 Ag NCs-Hg^2+^ system in the presence of melamine with concentrations 1, 2, 4 and 5 μM, respectively). Reproduced from Du *et al.* (*138*) with permission from the Centre National de la Recherche Scientifique (CNRS) and The Royal Society of Chemistry. NC=nanocluster

Optical chemical sensors are devices which measure changes in optical phenomena – absorbance, reflectance, luminescence, *etc*. – caused by an interaction of the analyte with the recognition element. Colloidal silver is coloured, with pronounced absorbance/LSPR maxima, and this is often the basis of colourimetric sensor development. Recently, silver nanoclusters (AgNCs) have found use as the fluorescent species in luminescent sensors. A summary of the silver nanomaterial-based optical sensors for food applications is presented in Table 2 (*42**,**43**,**46**,**101**-**139*). Selected examples of optical chemical sensors, both colourimetric and fluorescent, are shown in Fig. 4 (*113*, *138*).

**Table 2:** Optical chemical sensors based on silver nanomaterials

Analyte	Sample	Analytical method	Recognition element	Nanomaterial	LOD	Ref.
VOC from decomposition	Banana	Colourimetry	Direct - AgNPs	AgNPs	–	(*101*)
VOC from decomposition	Onion	Colourimetry	Direct - AgNPs	AgNPs (PEG stabilised)	–	(*102*)
Trimethylamine	Buffer	Colourimetry/LSPR	Resorcinol monoacetate	Au@Ag nanorods (*in situ*) in agarose hydrogel	8.6 nM	(*103*)
Hydrogen sulfide	Chicken, carp	Colourimetry	Direct - AgNPs	AgNPs (gellan gum stabilised)	0.81 µM	(*104*)
Ammonia	Fish, meat	Colourimetry/LSPR	Direct - AgNPs	AgNPs in bacterial nanopaper	0.574·10**^–^**^6^ g/mL	(*105*)
Sugars	Soft drinks and apple	Colourimetry	Direct - AgNPs	AgNPs (*in situ*, CTAC stabilised)	8.7 µM	(*106*)
Flavonoids (4 types)	Buffer	Colourimetry	Direct - AgNPs	AgNPs (*in situ*, PVP stabilised)	0.03–0.1 µg/mL	(*107*)
Lysozyme	Milk	Colourimetry/LSPR	Direct - AgNPs	AgNPs (glutamic acid stabilised)	1.5 nM	(*108*)
Caffeine	Tea, Coca-Cola	Colourimetry	AgNPs (MMIP extraction)	AgNPs	1 µg/L	(*109*)
Gamma-aminobutyric acid	Green tea	Second-order light scattering	AgNPs (SI pretreatment)	AgNPs	39.6 mg/L	(*110*)
Antioxidants (20 types)	Tea, lemon	Colourimetry	Direct – AgNPs/AuNPs	AgNPs and AuNPs (multiple stabilisers)	3.5–47 nM (7 types)	(*111*)
Se(IV)	Mushroom garlic	LSPR/colourimetry	Direct – Ag nanoprisms	Ag nanoprisms	1.2 µg/L	(*112*)
Chloride	Water	Colourimetry, distance based	AgNPs and H_2_O_2_, reaction	AgNPs	2 mg/L	(*113*)
Hg(II)	Buffer	Colourimetry	Direct – AgNP reaction	AgNPs (gelatin stabilised)	25 nM	(*114*)
Hg(II)	Water, milk	LSPR/colourimetry	Direct – Ag-AuNPs	Ag–AuNPs	5 nM	(*115*)
Cu(II)	Water	Colourimetry/LSPR	Cu(II) catalysed reaction	AgNPs (starch stabilised)	0.24 µg/L	(*116*)
Cu(II)	Water, tomato, rice	Colourimetry	Cu(II) catalysed reaction	AgNPls (CTAB stabilised)	0.3 µg/L	(*117*)
Fe(II)	Fe suppl.	Colourimetry	Ag^+^ reduction	AgNCs (PMAA stabilised)	76 nM	(*46*)
Carbendazim	Water, apple, carrot	Colourimetry	ABT	AgNPs (ABT stabilised)	1.04 µM	(*118*)
Triazophos	Water, rice, apple	Colourimetry	MPA and GAA	AgNPs (MPA and GAA stabilised)	0.08 μM	(*119*)
Thiophanate-methyl	Water, tomato	Colourimetry	Direct - AgNPs	AgNPs (citrate stabilised)	0.12 μM	(*120*)
Diazinon	Fruit, vegetable	Colourimetry	Direct - AgNPs	AgNPs (borohydride stabilised)	7 µg/L	(*121*)
Malathion	Water, apple	Colourimetry	Aptamer	AgNPs (citrate stabilised)	0.5 pM	(*122*)
Methamidophos, malathion	Water	Colourimetry	Enzyme (inhibition)	Au@AgNPs	0.17 nM 0.11 nM	(*123*)
Melamine	Milk	Colourimetry/LSPR	Direct - AgNPs	AgNPs (green synthesised)	2 µM	(*42*)
Hydrogen peroxide	Chicken	LSPR/colourimetry	Ag oxidation	Au@AgNRs	3.2 µM	(*124*)
*L. monocytogenes*	Pork	Colourimetry	Antibody, aptamer	Antibody modified AgNCs	10 CFU/mL	(*125*)
Ochratoxin A	Flour, beer	Fluorescence	Aptamer	AgNPs	8.7 nM	(*126*)
Ochratoxin A, aflatoxin	Rice, corn, wheat	Fluorescence	Aptamer	AgNCs (*in situ*)	0.2 pg/mL OTA 0.3 pg/mL AFB_1_	(*127*)
T-2 mycotoxin	Oat, corn	Fluorescence	Aptamer	AgNCs (*in situ*)	0.03 pg/mL	(*128*)
T-2 mycotoxin	Wheat, maize	Fluorescence	Aptamer	AgNCs	0.93 pg/mL	(*129*)
Mycotoxins (5 types)	Wheat, nut, milk	Colourimetry	Direct – AgNP aggregation	AgNPs (caffeic acid, PVP or dopamine stabilised)	2.1 ng/mL to 7 ng/mL	(*130*)
*E. coli*	Milk, water	Fluorescence	DNAzyme	DNA-templated AgNCs	60 CFU/mL	(*131*)
*S. typhimurium*	Chicken meat	Fluorescence	Aptamer	AgNC (*in situ*)	50 CFU/mL	(*132*)
*P. aeruginosa*	Milk, juice	Fluorescence	Antibody	AgNP (glucose stabilised)	1.5 CFU/mL	(*133*)
Carbamate	Water, fruit	Fluorescence/ colourimetry	Enzyme inhibition	Rhodamine modified AgNPs	0.023 ng/L	(*134*)
Malathion	Water, fruits, vegetables	Fluorescence	Enzyme inhibition	AgNPs	0.556 fM	(*135*)
Kanamycin	Milk	Fluorescence	Aptamer	AgNCs	1 nM	(*136*)
Fe(III), thiosulfate	Fruits, yoghurt, rice	Fluorescence	Direct - AgNCs	AgNCs in PVA and borax hydrogel	0.045 and 0.060 µM	(*137*)
Melamine	Milk, formula, dog food	Fluorescence	Direct - AgNCs	AgNCs (PEI stabilized); in agarose hydrogel	30 nM	(*138*)
Nitrite	Water	Fluorescence	Direct - AgNCs	AgNCs (PEI stabilised)	100 nM	(*139*)
Nitrite	Sausages	Chemiluminescence	H_2_O_2_ reaction	Ag@AgCl@GO@Fe_3_O_4_	25 nM	(*43*)

LOD=limit of detection, VOC=volatile organic compound, AgNP=silver nanoparticle, PEG=polyethylene glycol, LSPR=localized surface plasmon resonance, CTAC=cetyltrimethylammonium chloride, PVP=polyvinylpyrrolidone, MMIP=magnetic molecularly imprinted polymeric microsphere, AuNP=gold nanoparticles, AgNPI=silver nanoplate, CTAB=cetyltrimethylammonium bromide, PMAA=polymethylacrylic acid, ABT=4-aminobenzenethiol, MPA=3-mercaptopropinonic acid, GAA=guanidine acetic acid, AgNR=silver nanorod, AgNC=silver nanocluster, PVA=polyvinyl alcohol, PEI=polyethyleneimine, GO=graphene oxide

### Colourimetric sensors

Colourimetric sensors (measuring changes in the absorption or reflectance during analysis) are the single most represented group of sensors in this review, possibly owing to their simplicity of fabrication and use. As such, they have found use for determination of food quality as well as food safety. In the scope of food quality determination, colourimetric sensors have been developed for evaluation of food composition, but also for food freshness monitoring (*e.g*. through detection of decomposition products). Examples of the latter include AgNP solutions which change colour (from red or yellow to transparent) when AgNPs bind to volatile decomposition products of bananas (*101*) or onions (*102*). While these sensors were simple, specialised measurements were necessary and analytical performance was not evaluated. On the other hand, Lin *et al*. (*103*) have developed colourimetric sensors for biogenic amines (represented by trimethylamine), which are generated by bacterial decarboxylation of amino acids in protein-rich foods. The sensors were developed by immobilising Au nanorods, AgNO_3_ and resorcinol monoacetate (RMA) within an agarose hydrogel. Biogenic amines cause hydrolysis of RMA; the product of this reaction reduces Ag^+^ to form Au@Ag core-shell nanorods, and the colour change of the hydrogels can be evaluated by a smartphone. Agarose hydrogels loaded with AgNPs were also used to detect H_2_S formed during chicken and carp spoilage (*104*). These sensors were based on a very stable Ag_2_S formation and the resulting colour change of the hydrogels. Ammonia vapour is another byproduct of fish/meat spoilage and its concentration was quantified using bacterial cellulose with embedded AgNPs (*105*). In this sensor, ammonia causes etching of the AgNPs, altering their population density, size distribution and interparticle distance; this in turn changes colour of the sensing paper.

The other important aspect of food quality control is assessment of the composition, *i.e*. contents of key ingredients. Some sensors exploit the reducing capabilities of these components and their potential to generate AgNPs *in situ* by reduction of Ag^+^ ions; the colour of the formed AgNPs is usually measured with a spectrophotometer. Examples of this approach include a simple and quick sensor for sugar content determination (combined monosaccharides, disaccharides or polyols) (*106*), as well as a sensor for determination of four different reducing flavonoids (*107*). On the other hand, some analytes cause aggregation of the already prepared silver nanoparticles, which in turn causes changes in their absorption spectra – this was the basis for a sensor for lysozyme in milk (*108*). This sensor was optimised by selecting glutamic acid as the ideal capping agent which was not subject to interference from several common milk components and a very low LOD (1.5 nM) was achieved. Since aggregation can be caused by multiple substances found in complex food matrices, Deng *et al*. (*109*) have coupled AgNPs with magnetic molecularly imprinted polymeric microspheres (MMIPs) to improve the selectivity towards caffeine. Although a much lower LOD of caffeine than needed for tea and Coca-Cola samples was obtained (1 µg/L), this sensor does include a previous extraction step. Sequential injection has also been used as pretreatment step, in a sensor for detecting γ-aminobutyric acid (GABA), a health-promoting substance found in certain foods (*110*). The positively charged GABA causes aggregation and a colour change of negatively charged AgNPs in solution. However, second-order light scattering was used for detection instead of colourimetry. Bordbar *et al*. (*111*) have developed a colourimetric array (an optoelectronic tongue), which can discriminate among 20 different antioxidants in food. Due to AgNPs, AuNPs and six different reducing/capping agents, the array provides a unique colourimetric response for each antioxidant: nanoparticle aggregation causes a red shift, while substitution of the capping agent with the antioxidant causes a change in the absorbance value. Very low LODs were thus achieved for seven antioxidants, while five antioxidants were detected simultaneously in tea and lemon juice. Different innovative approaches were used for the detection of selenium and chloride, two necessary elements which are undesirable at high concentrations. Selenite ions etch Ag nanoprisms and change their shape to nanodiscs, which causes an LSPR wavelength blue shift and colour change (*112*). Interestingly, this mechanism was confirmed by establishing that there was no effect on spherical AgNPs. The well-known oxidative etching of AgNPs by Cl^–^ in the presence of H_2_O_2_ was employed to develop a distance-based paper chloride sensor, Fig. 4a (*113*). In this simple and inexpensive sensor, the length of the white precipitate, as measured with a ruler, correlates with the concentration of chloride ions. Although coloured samples may present an issue, the naked eye LOD with water samples was 2 mg/L and no interference was observed from several ions (including halides).

Within the scope of food safety, colourimetric sensors have been developed for the detection of heavy metals, pesticides, bacteria and unwanted food additives. Heavy metals, ingested *via* food or water sources, pose a serious threat to human health. Mercury is one of the most toxic heavy metals and several sensors for its determination have been developed. In one example, gelatin-stabilised AgNPs were used for mercury sensing; these nanoparticles form an amalgam with Hg^2+^, which causes a change in the colour/spectrum (*114*). The nanoparticles were evaluated in three sensing forms: AgNPs in solution, in polyvinyl alcohol (PVA) hydrogels and on paper strips. While all sensors showed similar sensitivities, the response in the hydrogels and on paper was much slower, due to the heterogeneous reaction. Nevertheless, due to the simplicity, the paper strips were highlighted as having great potential for monitoring mercury in food samples. Tao *et al*. (*115*) achieved an improved LOD of Hg(II) by developing plasmonic sensors based on Ag–Au alloy nanoparticles (Ag–AuNPs). After the reaction with mercury, the amalgam shell caused a blue shift of the LSPR peak. Again, in addition to the colloid, heterogeneous sensors were developed: Ag–AuNPs were immobilised on an indium tin oxide glass surface using PDDA as a binder or embedded within a PVA film. Interestingly, the plasmonic sensor based on PVA demonstrated the best sensitivity and was used to detect mercury in drinking water and milk.

In the case of Cu(II) sensors, a different detection mechanism was used: catalytic etching of Ag by thiosulfate in the presence of Cu^2+^ ions. This etching causes a colour change of a starch-stabilised AgNP suspension, correlated with Cu(II) concentration (*116*). Chaiyo *et al.* (*117*) went a step further and immobilised Ag nanoplates (AgNPls) on paper substrates to form simple paper-based devices relying on the same mechanism. An LOD similar to suspension sensors (0.3 µg/L) was achieved with semi-quantitative image processing, but the number of samples was extended to include tomato and rice. The device holds great potential for portable, rapid, simple and low-cost field testing. Deficiency of iron is a common health problem, but the intake of this heavy metal *via* food or supplements should also be controlled. A colourimetric Fe(II) sensor was based on the growth of silver nanoclusters (AgNCs) upon reduction of excess Ag^+^ in the solution (*46*). While the fluorescence property of AgNCs is commonly exploited, this is a rare example of measurement of the size-dependent colour change of the clusters.

Pesticides represent a major group of toxins detected with colourimetric sensors. Non-covalent bonding between the pesticide and the nanoparticle stabiliser molecule is the basis of several sensors. In one example, 4-aminobenzenethiol (ABT)-functionalised silver nanoparticles act as a colourimetric probe for the fungicide carbendazim (*118*). Due to strong ion-pair and π-π interactions between the stabiliser molecule and analyte, intense aggregation occurs (large conjugate network formation), which causes a visible red shift of the solution absorbance. The same group expanded on this sensing mechanism and developed AgNPs bifunctionalised with 3-mercaptopropinonic acid (MPA) and guanidineacetic acid (GAA) (*119*). These nanoparticles can selectively bind triazophos (a broad spectrum organophosphorus pesticide) *via* hydrogen bonding; further aggregation *via* π-π interactions causes and even larger red shift than in their previous example and enables achieving low LODs (0.08 µM). Furthermore, the sensor did not react with several interferents and mixtures thereof (including cations, anions and other pesticides). In other examples, hydrogen bonding between citrate-stabilised AgNPs and thiophanate-methyl was exploited (*120*), as well as the non-covalent interaction between borohydride-stabilised AgNPs and diazinon pesticide (*121*). In addition to these non-covalent interactions, pesticides were detected with biosensors using biological recognition elements (aptamers and enzymes), which ensures high selectivity and, as demonstrated, superior LODs. The lowest LOD for pesticides (0.5 pM) was achieved by Bala *et al.* (*122*) with their aptasensor for malathion. In this solution-based sensor, the key components are AgNPs, a basic hexapeptide and malathion-specific aptamer. With no malathion present, the aptamer binds to the peptide and does not affect the optical properties of AgNPs. However, if the aptamer reacts with malathion in the sample, then the free peptide interacts with negatively charged AgNPs, causing their aggregation and colour change. Enzyme inhibition was the basis for another biosensor for organophosphorus pesticides (methamidophos and malathion) (*123*). Alkaline phosphatase (ALP) catalyses the dephosphorylation of the added substrate *p*-aminophenyl phosphate (*p*-APP); the product reduces Ag(I) ion to Ag which is spontaneously deposited on the surface of the present AuNPs to form Au@AgNPs. Superiority of Au@AgNPs, compared to pure AgNPs or AuNPs, was demonstrated and sub-nanomolar LODs were obtained for both pesticides. A drawback may be the 2-hour enzyme inhibition period and an additional 30-minute period for Ag reduction and growth.

Colourimetric sensors were developed for several harmful food additives. Melamine, an illegal adulterant for presenting higher protein content of food, was detected with green-synthesised AgNPs, which aggregate upon analyte interaction and change colour (*42*). Since hydrogen peroxide is commonly used for oxidation and bleaching, a colourimetric sensor for its detection in food has also been developed (*124*). This sensor uses Au@Ag nanorods (Au@Ag NRs). In the presence of H_2_O_2_, silver atoms on the surface of the nanorods are oxidised to silver ions, thereby resulting in an LSPR red shift and colour change. Lastly, in an example of colourimetric bacteria detection, AgNCs play the role of an artificial enzyme (*125*). IgY antibody-coated AgNCs bind to *Listeria monocytogenes*, which is captured by aptamer-modified magnetic beads and then the whole sandwich-type immunocomplex is transferred to the reporting system. The reporting system contains AuNPs and *o*-phenylenediamine (OPD), which causes their aggregation. The AgNCs catalyse OPD oxidation and cause deaggreagation of AuNCs with a pronounced effect on the UV-Vis spectrum. While the measurement procedure may seem complex, the assay takes 1 h and can specifically detect the target bacteria with a low detection limit (10 CFU/mL).

### Luminescent sensors

Fluorescence measurements enable greater sensitivity and lower detection limits than obtainable with colourimetric sensors. As such, the fluorescent sensors found in this survey were developed exclusively for food safety monitoring – where detection of the smallest amounts of harmful agents is needed. This is best demonstrated in the case of sensors for mycotoxins, toxic fungal metabolites harmful to both humans and livestock. For example, a quick fluorescent method for detecting ochratoxin A (OCH) in flour and beer has been developed (*126*). This FRET (Förster resonance energy transfer) sensor was based on nitrogen-doped carbon dots (CD) as energy donor and AgNP modified with aptamer and 6-mercapto-1-hexanol (MCH) as energy acceptor. Upon interaction of OCH with the aptamer on AgNPs, FRET is inhibited and the resulting fluorescence intensity can be correlated with OCH concentration in the sample. The sensor has a wide concentration range (10–5000 nM) and can detect OCH in 30 min due to the use of MCH as stabiliser. Zhang *et al*. (*127*) developed another fluorescent aptasensor that further improved the sensitivity and provided detection limits of ochratoxin A and aflatoxin even lower than the voltammetric sensors previously described. Upon aptamer-mycotoxin interaction, signal probes are released into the supernatant and used as scaffolds for *in situ* fluorescent silver nanocluster (AgNC) synthesis. Fluorescence intensity of AgNCs was further increased with Zn^2+^ ions, achieving LODs of 0.2 pg/mL for OCH and 0.3 pg/mL for aflatoxin, respectively. A similar approach with fluorescent AgNCs has been adopted to develop aptasensors for another mycotoxin – T-2 mycotoxin. In one example, the DNA remaining after competitive binding of T-2 to the aptamer is amplified *via* exponential isothermal amplification reaction (EXPAR) (*128*). The produced DNA is used for *in situ* fluorescent AgNC formation and this provides a low concentration range (1 pg/mL to 100 ng/mL) and LOD (0.03 pg/mL). Alternatively, previously synthesised aptamer-functionalised AgNCs can be deposited on MoS_2_ nanosheets, which causes quenching *via* FRET; presence of the T-2 mycotoxin causes recovery of the fluorescence signal which can be correlated with analyte concentration (*129*). Mycotoxins can also be detected with a colourimetric system, although with a tradeoff in sensitivity. Recently, a paper-based six-sensor array has been fabricated for the simultaneous detection and discrimination of five different mycotoxins (*130*). Caffeic acid, PVP and dopamine were used as stabilisers of AgNPs and AuNPs, which aggregate upon interaction with mycotoxins causing a colour change. For example, caffeic acid-capped AgNPs are mostly aggregated by aflatoxins and ochratoxin. Established LODs for the five mycotoxins were in the range from 2.1 to 7 ng/mL, which is unfortunately several orders of magnitude higher than the fluorescent sensors.

Fluorescence was also the most represented analytical method for detection of bacteria in food and beverage samples. In an improvement over their colourimetric sensor (*140*), Zheng and Zhang (*131*) developed a fluorescent turn-on *E. coli* sensor. In the presence of *E. coli* lysate, the freed AChE is transferred into the DNA-AgNCs-containing system to catalyse the hydrolysis of acetylthiocholine (ATCh) to TCh. TCh significantly increases DNA-AgNCs fluorescence (*via* ligand-to-metal charge transfer (LMCT) or ligand-to-metal–metal charge transfer (LMMCT)), generating a 60 CFU/mL LOD. In a *Salmonella typhimurium* sensor, single-stranded sequences were released from an aptamer-sequence complex upon recognition, initiating a branch migration to release complementary scaffolds for AgNCs (*132*). This amplification was repeated thrice, producing highly fluorescent AgNCs *in situ* and achieving ultrasensitive (LOD=50 CFU/mL) and very linear bacterial detection. This lowest LOD (1.5 CFU/mL) was achieved with a *Pseudomonas aeruginosa* sensor based on a pyrimidine derivative probe tagged with glucose-stabilised AgNPs (*133*). The green-synthesised sensor was functional in several samples, including water, soil, milk, sugarcane and orange juices.

Among pesticides, fluorescent sensors for carbaryl (carbamate) (*134*) and malathion (organophosphorus) (*135*) were developed. The carbaryl sensor relies on the fact that carbamate pesticides inhibit the activity of acetylcholinesterase (*134*). Adsorption of rhodamine on AgNP causes quenching of its fluorescence and this can be undone by thiocholine (along with aggregation-induced colour changes). With inhibited acetylcholinesterase, less thiocholine is produced, and the reduction in fluorescence intensity can be correlated with pesticide concentration. The fluorescent assay is more sensitive than the colourimetric one and produces LOD an order of magnitude lower. The same strategy, although without a fluorophore and instead relying on the fluorescence of AgNPs, was used for detecting the organophosphorus pesticide, malathion (*135*). DNA-templated AgNCs were also used as energy donor and AuNPs as energy acceptor in a surface plasmon-enhanced energy transfer (SPEET) sensor for the antibiotic kanamycin in milk samples (*136*). In an example of heterogeneous sensing, it was found that polyvinyl alcohol (PVA) and borax hydrogel can be used both as a reducing agent and immobilisation matrix for the formation of fluorescent AgNCs (*137*). This hydrogel acts as a dual on-off sensor for Fe(III) and thiosulfate. As Du *et al.* (*138*) demonstrated, fluorescence of AgNCs stabilised by polyethyleneimine (PEI) is quenched by Hg^2+^, but recovered by melamine, Fig. 4b. This was the basis for the development of a fluorescent sensor for this harmful food additive. The AgNCs were further embedded within an agarose hydrogel to obtain simple devices for field testing. PEI-stabilised AgNCs were also used as a sensor for nitrite (*139*). In this sensor, the product of nitrite and added hydrogen peroxide reaction causes aggregation and quenching of AgNCs; a 100 nM LOD was achieved.

In the only example of a chemiluminescent sensor, nitrite was detected starting with Ag@AgCl nanoparticles grafted onto graphene oxide@Fe_3_O_4_ nanocomposite (*43*). This composite causes light-initiated hydrogen peroxide generation and the peroxide reacts with nitrite in the sample. Decomposition of the product and uranine excitation causes chemiluminescence, the intensity of which is proportional to nitrite concentration. The obtained LOD surpassed other optical sensors found in this review.

## CONCLUSIONS AND OUTLOOK

Silver nanoparticles have demonstrated the ability to significantly enhance detection capabilities of analytical devices and have accordingly been used in the development of electrochemical and optical chemical sensors. Among the papers analysed in this review, silver nanoparticles are commonly obtained by reduction of the silver nitrate precursor using borohydride, citrate or plant-derived reducing agents. A multitude of different stabilisers has been explored, as best evidenced from the previous chapters. The choice of stabiliser is of paramount importance, since it dictates the size and shape of the nanoparticles (which has an immense effect on the nanomaterial and sensor properties), but it is also in some sensors responsible for the analyte recognition itself.

We have analysed a total of 81 different sensors in this review. The majority of analytes were those related to food safety: most sensors were developed for detection of food additives and drugs (almost 24%), pesticides (20%), microorganisms (11%), mycotoxins (10%), and fertilisers and heavy metals with 7% in each category. On the other hand, 10% of all sensors were for food composition determination and 11% were for freshness indicators, such as decomposition products. Regarding the transduction mechanism, we see that the sensors are almost evenly divided into electrochemical (39/81) and optical (42/81) sensors. Among both classes of sensors, generally simpler and quicker analytical methods are dominating the field: most electrochemical sensors relied on voltammetric techniques (32/39 electrochemical sensors), while colourimetry was most pronounced among optical sensors (28/42 optical sensors). Nevertheless, more complex techniques were used where achieving low limits of detection was of principal importance for food safety. Fluorescent sensors are generally much more sensitive than their colourimetric counterparts and femtomolar LODs were achieved for some mycotoxins and pesticides. Similarly, electrochemical impedance spectroscopy enabled detection of certain pesticides and drugs down to impressive femto- or even attomolar concentrations.

Regardless of the material used, in the development of electrochemical sensors, bare working electrodes are not the optimal choice due to their slow electron transfer kinetics. The oxidative peak of silver is about 100-fold more intense than the signal of colloidal gold with the same particle diameter and concentration. Thereby, the role of AgNPs in the fabrication of food sensors is directed towards amplification of sensitivity, while selectivity is improved by using aptamers or MIPs. To obtain faster electron transport, silver nanoparticles can be coated onto the working electrode surface: directly or *via* a polymeric layer. However, AgNPs are usually combined with other nanomaterials to form hybrid architectures. Hence, AgNPs are deposited onto SWCNTs, MWCNTs, MoS_2_ nanosheets, graphene, graphene oxide, reduced graphene oxide, *etc*. There are examples of silver nanomaterial-modified screen-printed electrodes, carbon paste electrodes, solid amalgam electrodes, disposable PVC electrodes and inkjet-printed flexible sensors. A major advantage of electrochemical sensors is the possibility of analysis of turbid samples, which most food samples are, without extensive sample pretreatment.

Optical (bio)chemical sensors are being thoroughly developed since they offer numerous advantages such as simplicity of fabrication and use, as well as lower cost. Optical sensing with AgNPs is commonly based on the following mechanisms: Ag(I) reduction by analyte for *in situ* generation of AgNPs/AgNCs with specific optical properties (colour, fluorescence, LSPR maxima); chemical reaction of the Ag nanomaterial causing change in optical properties due to shape/size transformation or formation of a coloured reaction product; interaction of the AgNP stabiliser molecule with the analyte which causes aggregation/deaggregation of AgNPs and a subsequent colour change; catalytic effect of AgNCs on the detection reaction; fluorescent AgNCs formed *in situ* using (DNA) scaffolds released during a sensing event; and AgNCs acting as FRET or LMCT energy acceptors/quenchers (specifically in fluorescent sensors). However, most optical sensors detected in this survey were developed for homogeneous sensing in solutions, which makes reuse of the reagents (silver nanoparticles) difficult, driving the price up. Only several examples of optical sensors were found where the AgNPs were immobilised, usually in the following matrices: paper, bacterial nanocellulose, PVA and agarose hydrogels. Sensible immobilisation of the optical sensing chemistries into thin films or hydrogels would bring these sensors a step towards mobile, reusable and/or continuous food quality/safety monitoring and ultimately commercial products (*141*). Until then, electrochemical sensors have an advantage for this particular application.

We can see that nanosensing is still a growing field with many possibilities for both food safety and quality sectors, albeit with remaining challenges towards successful commercialisation. While green AgNP synthetic routes are already being promoted, future breakthroughs may come from leveraging greener, versatile and high-throughput technologies for fabrication of solid-state nanoparticle-based sensors, such as screen printing or inkjet printing (*142*). Together with the observed trends in miniaturisation and automation, smart sensors (and smart packaging) were labelled as the future of the food industry. Integration with low-cost mobile electronics or smartphones would enable simple portable on-site sensing, while incorporation of wireless communication would provide networking, improved connectivity and a higher degree of automation (*143*). Successful integration of these cutting-edge technologies, along with simultaneous advances in analytical performance of nanoparticle-based devices, will bring about this next generation of food safety and quality sensors.
